# Distribution of prostate nodes: a PET/CT-derived anatomic atlas of prostate cancer patients before and after surgical treatment

**DOI:** 10.1186/s13014-016-0615-9

**Published:** 2016-03-11

**Authors:** Nina-Sophie Hegemann, Vera Wenter, Sonja Spath, Nadia Kusumo, Minglun Li, Peter Bartenstein, Wolfgang P. Fendler, Christian Stief, Claus Belka, Ute Ganswindt

**Affiliations:** Department of Radiation Oncology, Klinikum der Universität, Ludwig-Maximilians-University (LMU), Marchioninistrasse 15, 81377 Munich, Germany; Department of Nuclear Medicine, Klinikum der Universität, Ludwig-Maximilians-University (LMU), Marchioninistrasse 15, 81377 Munich, Germany; Department of Urology, Klinikum der Universität, Ludwig-Maximilians-University (LMU), Marchioninistrasse 15, 81377 Munich, Germany

**Keywords:** Prostate cancer, Lymph node metastases, Anatomic atlas, Choline PET/CT

## Abstract

**Background:**

In order to define adequate radiation portals in nodal positive prostate cancer a detailed knowledge of the anatomic lymph-node distribution is mandatory. We therefore systematically analyzed the localization of Choline PET/CT positive lymph nodes and compared it to the RTOG recommendation of pelvic CTV, as well as to previous work, the SPECT sentinel lymph node atlas.

**Methods:**

Thirty-two patients being mostly high risk patients with a PSA of 12.5 ng/ml (median) received PET/CT before any treatment. Eighty-seven patients received PET/CT for staging due to biochemical failure with a median PSA of 3.12 ng/ml. Each single PET-positive lymph node was manually contoured in a “virtual” patient dataset to achieve a 3-D visualization, resulting in an atlas of the cumulative PET positive lymph node distribution. Further the PET-positive lymph node location in each patient was assessed with regard to the existence of a potential geographic miss (i.e. PET-positive lymph nodes that would not have been treated adequately by the RTOG consensus on CTV definition of pelvic lymph nodes).

**Results:**

Seventy-eight and 209 PET positive lymph nodes were detected in patients with no prior treatment and in postoperative patients, respectively. The most common sites of PET positive lymph nodes in patients with no prior treatment were external iliac (32.1 %), followed by common iliac (23.1 %) and para-aortic (19.2 %). In postoperative patients the most common sites of PET positive lymph nodes were common iliac (24.9 %), followed by external iliac (23.0 %) and para-aortic (20.1 %). In patients with no prior treatment there were 34 (43.6 %) and in postoperative patients there were 77 (36.8 %) of all detected lymph nodes that would not have been treated adequately using the RTOG CTV. We compared the distribution of lymph nodes gained by Choline PET/CT to the preexisting SPECT sentinel lymph node atlas and saw an overall good congruence.

**Conclusions:**

Choline PET/CT and SPECT sentinel lymph node atlas are comparable to each other. More than one-third of the PET positive lymph nodes in patients with no prior treatment and in postoperative patients would not have been treated adequately using the RTOG CTV. To reduce geographical miss, image based definition of an individual target volume is necessary.

## Background

Treatment strategies for high risk or nodal positive prostate cancer (PCA) remain ill defined. Different scenarios may be distinguished: Treatment of lymphatic pathways during radiotherapy in high risk, pN+ or cN+ PCA patients or treatment of isolated secondary lymphatic relapses. In order to define adequate radiation portals for any of the given scenarios a detailed knowledge of the lymph-node distribution is mandatory. In previous work, single photon emission computed tomography (SPECT) sentinel lymph node data were used to develop a realistic anatomic atlas [1–3]. However a shortcoming of any sentinel lymph node based atlas is the fact that only the putative drainage pathways are analyzed. The real distribution of pathologically affected lymph nodes is still not visualized. Until the introduction of PET-based imaging, the quality (sensitivity and specificity) of lymph-node diagnostics in PCA had been limited. Choline PET/CT offers a sensitivity and specificity of 84 % (95 % CI, 68–93 %) and 79 % (95 % CI, 53–93 %) in staging patients with yet untreated prostate cancer [4]. In restaging patients with biochemical failure Choline PET/CT has an even higher sensitivity and specificity of 85 % (95 % CI, 79–89 %) and 88 % (95 % CI, 73–95 %) [4]. In order to overcome the inherent limitation of the sentinel lymph node atlas mentioned above, we analyzed the anatomical distribution of Choline PET/CT positive lymph nodes in untreated patients and patients after radical prostatectomy. We compared this anatomic atlas to the RTOG recommendation of pelvic CTV [5] as well as to the SPECT sentinel lymph node atlas.

## Methods

### Data acquisition and patients’ characteristics

Patients were selected from a single-center database of 1191 F-18-Fluoroetyhlcholine (18F-FEC) or C-11-Choline PET scans performed from 2004 to 2012 at the Department of Nuclear Medicine, Ludwig-Maximilians-University Hospital Munich. One hundred and twenty-eighth patients were identified with PET positive lymph nodes and were available for follow-up. Patients who had simultaneously a hematogenous spread of cancer or PET positive lymph nodes above the diaphragm were excluded. One hundred and twelve of the 128 identified patients had a F-18-Fluoroetyhlcholine and 16 patients had a C-11-Choline PET/CT, as over time the tracer used had been changed from C-11-Choline to F-18-Fluoroethylcholine. Thirty-two patients received PET/CT before any surgical or radiotherapy treatment, hence with completely intact lymphatic pathways. Most of these patients (*n* = 26) (Table 1) were high risk patients according to the risk group definition of D’Amico [6] and in five of these patients androgen deprivation therapy had been started before PET/CT had been performed. PSA at the time of PET/CT in this group was 12.5 ng/ml (median). Ninety-six patients received PET/CT for staging due to PSA relapse after primary treatment: 87 patients postoperatively, 8 patients after definitive radiotherapy and 1 patient after high-intensity focused ultrasound (HIFU). All patients with radical prostatectomy as primary treatment received lymphadenectomy at the time of radical prostatectomy: In mean 12.8 lymph nodes were removed and 1.5 of these lymph nodes (mean) were pathologically proven as metastases. As the lymphatic pathways of patients with definitive radiotherapy and HIFU were not surgically changed before receiving a PET/CT, these patients were excluded from generating an anatomic atlas for patients with PSA relapse after initial curative treatment. Postoperative patients (Table 2), who received a PET/CT due to PSA relapse, had in median a PSA of 3.12 ng/ml before PET/CT. None of these patients had ongoing androgen deprivation therapy at the time of PET/CT. Fifty-eight patients of this group had a locally advanced tumor (≥pT3a) and 30 patients had evidence of pathologic lymph nodes (pN1) at radical prostatectomy. Thus this PET/CT derived anatomic atlas is based on 32 patients with no prior treatment and on 87 patients after radical prostatectomy and lymphadenectomy.

**Table 1:** Patients’characteristics – patients with no prior treatment (*n* = 32)

Characteristic	n (%)
Tumor stage	
cT1c	6 (18.8)
cT2a	1 (3.1)
cT2b	3 (9.4)
cT2c	4 (12.5)
cT3a	5 (15.6)
cT3b	3 (9.4)
Tx	6 (18.8)
cN1	32 (100)
cM0	30 (93.75)
cM1a	1 (3.1)
cM1b	0
D’Amico risk group	
Low	2 (6.3)
Intermediate	4 (12.5)
High	26 (81.25)
Initial PSA level (ng/ml)	
Mean	32.78
Median	21.71
Range	5.1–198.0
PSA at the time of PET/CT (ng/ml)	
Mean	22.7
Median	12.5
Range	0.03–99.0
Gleason score	
≤6	7 (21.88)
7 (3 + 4)	3 (9.4)
7 (4 + 3)	4 (12.5)
8	1 (3.1)
9	8 (25.0)
missing	9 (28.1)
Age (years)	
Mean	69.2
Median	71
Range	51–80
Time from initial diagnosis until PET/CT (months)	
Mean	10.8
Median	1
Range	0–83

**Table 2:** Patients’characteristics – postoperative patients (*n* = 87)

Characteristic	n (%)
Tumor stage	
pT2a	6 (6.9)
pT2b	4 (4.6)
pT2c	15 (17.2)
pT3a	20 (23)
pT3b	37 (42.5)
pT4	1 (1.1)
Tx	4 (4.6)
pN0	54 (62.1)
pN1	30 (34.5)
pM1a	3 (3.4)
R0	39 (44.8)
R1	41 (47.1)
Rx	7 (8.0)
Gleason score	
≤6	10 (11.5)
7 (3 + 4)	18 (20.7)
7 (4 + 3)	16 (18.4)
8	12 (13.8)
9	21 (24.1)
10	1 (1.1)
missing	9 (10.3)
Initial PSA (ng/ml)	
Mean	28.7
Median	10.35
Range	1.56–392.00
Nadir (postoperative PSA) (ng/ml)	
Mean	1.85
Median	0.23
Range	0–32.00
PSA at the time of PET/CT (ng/ml)	
Mean	6.43
Median	3.12
Range	0.21–56.00
Age (years)	
Mean	62.4
Median	63
Range	41–82
Time from surgery until PET/CT (months)	
Mean	48.2
Median	32.0
Range	0–209

### PET/CT imaging

Whole body PET/CT images extending from the base of the skull to the mid-thigh were acquired using a GE Discovery PET/CT 690, a Siemens Biograph 64 TruePoint PET/CT (Siemens Medical Solutions) or a Philips Gemini PET/CT scanner (Philips Medical Systems). Emission scans were initiated 60 min following intravenous administration of approximately 300 MBq 18F-FEC (*n* = 106 patients) or 5 min after administration of approximately 500 MBq C11-Choline (*n* = 13 patients). Diagnostic CT scans (100-190mAs, depending on the scanned organ region, 120 kV) were acquired with intravenous injection of iodine-containing contrast agent (Ultravist 300, Schering; or Imeron 300, Bracco; 2.5 mL/s) at a dose adjusted for body weight. Initiation of CT acquisition was delayed 50 s after injection of the contrast agent in order to depict the portal venous phase. Lymph nodes with pathologically increased tracer accumulation distinctly in excess of physiologic uptake were identified as positive nodes.

### Atlas of lymph node distribution

**Fig. 1:**
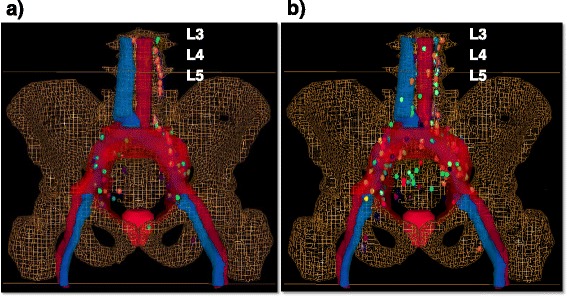
**a** Distribution and geographic miss according to RTOG – CTV (*red*) of PET positive lymph nodes in patients with no prior treatment (*n* = 32); **b** Distribution and geographic miss according to RTOG – CTV (*red*) of PET positive lymph nodes in postoperative patients (*n* = 87)

To allow a systematic topographic mapping of the lymph node locations, the cross-sectional nodal atlas published by Martinez-Monge et al. was used [7]. For each patient the number and location of the affected, PET-positive lymph nodes were documented. Beyond summarizing these data in a table, each single PET-positive lymph node was manually contoured in a “virtual” patient dataset to achieve a 3-D visualization of the cumulative PET positive lymph node distribution (Fig. 1a and b). The Oncentra MasterPlan (Version 4.3, Elekta, Crawley, UK) planning system was used for contouring and generating 3-D images for the atlas. Moreover, the PET-positive lymph node location in each patient was assessed with regard to the existence of a potential geographic miss (i.e. PET-positive lymph nodes that would not have been treated adequately by the RTOG consensus on clinical target volume definition of pelvic lymph nodes [5]).

## Results

### Location and distribution of PET positive lymph nodes

PET/CT images of each single PET positive lymph node were available as a 3-D reconstruction with individual coronal, axial and sagittal sections. During the systematic topographic evaluation of the lymph node locations, using the cross-sectional atlas published by Martinez-Monge [7], we observed a wide inter-individual variety of anatomic conditions (namely, the correlation between bone structures and vessel/bifurcation sites). To allow for a consistent definition of a lymph node location, we referred generally to the topographic sites of the vessel/bifurcation sites (not to bony landmarks as given by an axial section in computed tomography). All in all 287 PET positive lymph nodes were detected in all patients (Fig. 1a and b; Table 3). With an incidence of ≥10 % PET positive lymph nodes were seen in the area of external iliac (73 lymph nodes; 25.4 %), common iliac (70 lymph nodes; 24.4 %), para-aortic (57 lymph nodes; 19.9 %) and internal iliac (37 lymph nodes; 13.0 %). Since it is highly likely, that the surgical intervention has changed the lymphatic drainage, we analyzed the distribution of PET positive lymph nodes in postoperative patients and in patients with no prior treatment separately. Thus 78 PET positive lymph nodes were detected in patients with no prior treatment (Fig. 1a) and 209 PET positive lymph nodes were seen in postoperative patients (Fig. 1b). The detailed distribution and number of PET positive lymph nodes is shown in Table 4 for patients with no prior treatment and in Table 5 for postoperative patients. The number of positive lymph nodes per region ranged from 0 to 25 in patients with no prior treatment (median three lymph nodes) and from 0 to 52 in postoperative patients (median seven lymph nodes). The most common sites of PET positive lymph nodes in patients with no prior treatment were external iliac (25 lymph nodes; 32.1 %), followed by common iliac (18 lymph nodes; 23.1 %) and para-aortic (15 lymph nodes; 19.2 %). Fewer or no PET positive lymph nodes were found internal pudendal, rectal inferior/superior, at the periprostatic lymphatic plexus, at the seminal vesicle lymphatic plexus and sacral.

**Table 3:** Number and location of PET positive lymph nodes in all patients (*n* = 119)

Location	Region affected	Lymph nodes affected n; (%/all pathologic lymph nodes)	Geographical miss (*n* = regions)	Geographical miss n; (%/pathologic lymph nodes)
Internal pudendal nodes	7	7 (2.4)	0	0
Inferior rectal nodes	0	0		
Periprostatic lymphatic plexus	1	1 (0.3)	0	0
Seminal vesicle lymphatic plexus	0	0		
Perirectal lymphatic plexus	10	11 (3.8)	8	9 (81.8)
Perivesical lymphatic plexus	10	11 (3.8)	7	7 (63.6)
Sacral nodes	3	6 (2.1)	2	5 (83.3)
Internal iliac nodes	32	37 (13.0)	0	0
External iliac nodes	60	73 (25.4)	7	7 (9.6)
Superior rectal nodes	2	2 (0.7)	2	2 (100)
Common iliac nodes	52	70 (24.4)	11	12 (17.1)
Para-aortic nodes	31	57 (19.9)	31	57 (100)
Inguinal nodes	11	12 (4.2)	11	12 (100)
Total	219	287	79	111 (38.7)

**Table 4:** Number and location of PET positive lymph nodes in patients with no prior treatment (*n* = 32)

Location	Region affected	Lymph nodes affected n; (%/all pathologic lymph nodes)	Geographical miss (*n* = regions)	Geographical miss n; (%/pathologic lymph nodes)
Internal pudendal nodes	2	2 (2.6)	0	0
Inferior rectal nodes	0	0		
Periprostatic lymphatic plexus	1	1 (1.3)	0	0
Seminal vesicle lymphatic plexus	0	0		
Perirectal lymphatic plexus	3	4 (5.1)	3	4 (100)
Perivesical lymphatic plexus	3	3 (3.8)	3	3 (100)
Sacral nodes	0	0		
Internal iliac nodes	7	7 (8.9)	0	0
External iliac nodes	20	25 (32.1)	4	4 (16.0)
Superior rectal nodes	0	0		
Common iliac nodes	12	18 (23.1)	4	5 (27.8)
Para-aortic nodes	7	15 (19.2)	7	15 (100)
Inguinal nodes	3	3 (3.9)	3	3 (100)
Total	58	78	24	34 (43.6)

**Table 5:** Number and location of PET positive lymph nodes in postoperative patients (*n* = 87)

Location	Region affected	Lymph nodes affected n; (%/all pathologic lymph nodes)	Geographical miss (*n* = regions)	Geographical miss n; (%/pathologic lymph nodes)
Internal pudendal nodes	5	5 (2.4)	0	0
Inferior rectal nodes	0	0		
Periprostatic lymphatic plexus	0	0		
Seminal vesicle lymphatic plexus	0	0		
Perirectal lymphatic plexus	7	7 (3.3)	5	5 (71.4)
Perivesical lymphatic plexus	7	8 (3.8)	4	4 (50)
Sacral nodes	3	6 (2.9)	2	5 (83.3)
Internal iliac nodes	25	30 (14.4)	0	0
External iliac nodes	40	48 (23.0)	3	3 (6.3)
Superior rectal nodes	2	2 (1)	2	2 (100)
Common iliac nodes	40	52 (24.9)	7	7 (13.5)
Para-aortic nodes	24	42 (20.1)	24	42 (100)
Inguinal nodes	8	9 (4.3)	8	9 (100)
Total	161	209	55	77 (36.8)

In postoperative patients the most common sites of PET positive lymph nodes were common iliac (52 lymph nodes; 24.9 %), followed by external iliac (48 lymph nodes; 23.0 %) and para-aortic (42 lymph nodes; 20.1 %). Fewer or no PET positive lymph nodes were found in this group rectal inferior/superior, at the periprostatic lymphatic plexus, at the seminal vesicle lymphatic plexus and sacral. Sixty-seven of the 87 postoperative patients (77.0 %) had a PSA at the time of Choline PET/CT above the optimal PSA cutoff of ≥1.05 ng/ml (median 4.02 ng/ml; range 1.10–56.0) [8]. In this group a high number of non-pelvic lymph node findings (42 PET-positive paraaortic nodes) was seen.

### PET/CT-derived atlas

In addition to summarizing the distribution of PET-positive lymph nodes in tables, each single lymph node and its relation to vessels, bone, rectum, bladder, prostate and seminal vesicles were manually contoured in a “virtual” patient dataset, resulting in a 3-D visualization (Fig. 1a and b). The maximum standardized uptake value (SUV max) of the positive lymph node findings was in patients with no prior treatment in median 3.9 (mean: 5.3, range: 1.8–16.9) and in postoperative patients in median 5.1 (mean: 6.6; range 1.4–26).

### Geographic miss

**Fig. 2:**
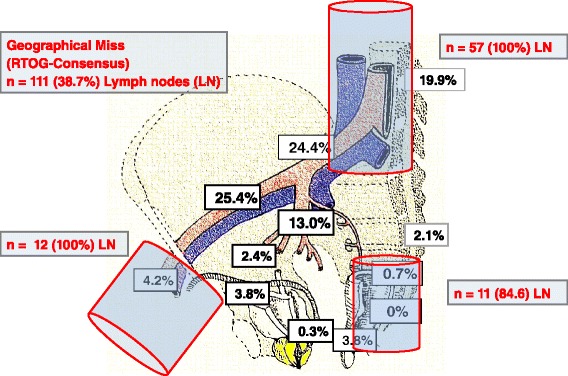
Schematic distribution of PET positive lymph nodes in all patients (*n* = 119) Anatomical regions and distribution (percentages in *white square frames*) of all detectable PET positive lymph nodes (*n* = 287). Main regions of geographical misses (*red*), when RTOG-Consensus (Lawton et al. [5]) for standard CTV delineation is used

A target volume according to the RTOG consensus of treatment of pelvic lymph nodes was further contoured in the “virtual” patient dataset and all positive lymph nodes findings were evaluated in regard to a potential geographic miss. Figure 1a and b show the cumulative PET-positive lymph node distribution of patients with no prior treatment and of postoperative patients with the RTOG CTV demonstrating a potential geographic miss. Figure 2 shows schematically the distribution of all PET-positive lymph nodes with the corresponding percentages of geographic miss. In patients with no prior treatment (*n* = 32) there were 24 regions with 34 lymph nodes involved that would not have been treated adequately using the RTOG clinical target volume definition for pelvic lymphatic drainage (“geographic miss”). In postoperative patients (*n* = 87) there were 55 of 161 (34.2 %) suspicious lymph node areas and 77 of all detected lymph nodes (36.8 %) that would not have been treated sufficiently. The detailed number, sites and proportions of geographic misses are shown in Tables 3, 4 and 5 and Figs. 1a, b and 2. Regarding the number of all detected PET-positive lymph nodes, the lymph node areas with the highest probability of geographic miss in patients with no prior treatment were perirectal and perivesical lymphatic plexus (100 %), para-aortic nodes (100 %) and inguinal nodes (100 %). In postoperative patients, superior rectal nodes (100 %), para-aortic nodes (100 %), inguinal nodes (100 %), sacral nodes (83.3 %) and perirectal lymphatic plexus (71.4 %) were not treated adequately by the RTOG CTV.

## Discussion

An awareness of the great variability of prostate cancer lymph node metastases is important for anyone who treats lymphogenous-metastasized prostate cancer. There are a few surgical and radiotherapy driven studies on mapping and extent of lymph node metastases in patients with newly diagnosed prostate cancer like the study of Heidenreich et al. [9], Joniau et al. [10], Ganswindt et al. [3] and Meijer et al. [11]. These studies aim to reduce the geographical miss of lymph node metastases while treating prostate cancer patients. They either performed an extended lymphadenectomy [9], or used for the clinical target volume the information of MR lymphography [12] or single photon emission computed tomography (SPECT) imaging [2] instead of applying the CTV as proposed by the RTOG consensus [5]. All of these studies were performed in patients with newly diagnosed prostate cancer, similarly to our 32 patients who received a Choline PET/CT as a primary staging. Our anatomic atlas based on Choline PET/CT as information for mapping prostate cancer lymph nodes is comparable to the anatomic atlases of Ganswindt et al. [3] and Meijer et al. [11]: the most common suspicious lymph node areas were found similarly to our study in the external, internal and common iliac and para-aortic regions. Unlike their studies there were fewer patients in our study who had PET-positive lymph node findings in the sacral or perirectal area. Similarly to the study of Meijer et al. who also applied the RTOG CTV to estimate a potential miss, we observed a geographical miss in the perirectal and perivesical lymphatic plexus and para-aortic nodes. To our best knowledge, there are no detailed mapping studies on prostate lymph node metastases using PET/CT imaging so far. Potentially this is the first Choline PET/CT derived atlas of prostate lymph node metastases in patients with no prior treatment. All three studies on the geographical distribution of lymph node metastases of patients with no prior treatment nevertheless show the need of an adequate imaging method before performing an individualized radiotherapy treatment of the lymphatic pathways with minimal geographical miss.

We do not know any other Choline PET/CT based anatomic atlas describing lymph node recurrences after radical prostatectomy together with pelvic lymphadenectomy. In Table 5 the wide variability of lymph node metastases with most lymph node findings in the external, internal and common iliac and para-aortic regions of lymphatic drainage is demonstrated. Many of these PET-positive lymph nodes would not be treated adequately by only applying the RTOG CTV. Nevertheless one has to say that this CTV has been developed for high-risk prostate cancer patients prior to radiotherapy as primary treatment and can therefore not be automatically applied to postoperative patients. Further one has to state, that 67 of the 87 postoperative patients (77.0 %) had a PSA at the time of Choline PET/CT well above the optimal PSA cutoff of ≥1.05 ng/ml (median 4.02 ng/ml; range 1.10–56.0). Thus it is not surprising that these patients had a high number of non-pelvic lymph node findings. The anatomic extent of lymph node involvement depends on well-known risk factors like Gleason score and PSA. Taking into account these factors, a Choline PET/CT should be performed before any irradiation in these high risk groups. Nevertheless the sole treatment of the PET-positive lymph nodes is not reasonable, because Choline PET/CT, as Tilki et al. showed [13], underestimates the true extent of affected lymph nodes. Tilki et al. performed a lymphadenectomy in patients with rising prostate specific antigen after radical prostatectomy and compared the histologic results with the findings of 18F-FEC PET/CT. They concluded that a positive 18F-FEC PET/CT result correctly predicts the presence of lymph node metastases in the majority of prostate cancer patients with biochemical failure after radical prostatectomy but does not allow for localization of all metastatic lymph nodes and therefore is not adequately accurate for the precise estimation of extent of nodal recurrence in these patients [13]. Therefore salvage lymphadenectomy and/or salvage radiotherapy due to rising PSA after primary curative radical prostatectomy should not only be performed in the region of the PET-positive lymph nodes but in the adjacent lymph node areas, as they might often be microscopically affected without a PET-positive signal. With prostate-specific membrane antigen (PSMA) PET/CT one expects to have an even better sensitivity and specificity in detecting affected lymph nodes [14]. Until now large numbers of lymph node positive prostate cancer patients diagnosed by PSMA PET/CT are missing and it remains questionable, whether the anatomic distribution of PET positive lymph nodes in prostate cancer will be changed by the usage of PSMA instead of Choline as a tracer.

## Conclusions

Choline PET/CT and SPECT sentinel lymph node atlases are comparable to each other. In both atlases describing patients prior to any treatment the most common suspicious lymph node areas were the external, internal and common iliac and para-aortic regions. Similarly, in postoperative patients most PET positive lymph node findings were in the external, internal and common iliac and para-aortic regions of lymphatic drainage. More than one-third of the PET positive lymph nodes in patients with no prior treatment and in postoperative patients would not have been treated adequately using the RTOG CTV. To reduce geographical miss, image based definition of an individual target volume is necessary. The extent of a possible geographic miss while using the RTOG CTV can be exemplarily seen in the present study.

### Ethics statement

This retrospective study was exempt from requiring ethics approval. Bavarian state law (Bayrisches Krankenhausgesetz/Bavarian Hospital Law §27 Absatz 4 Datenschutz (Dataprotection)) allows the use of patient data for research, provided that any person’s related data are kept anonymous. German radiation protection laws request a regular analysis of outcomes in the sense of quality control and assurance, thus in the case of purely retrospective studies no additional ethical approval is needed under German law.

### Consent for publication

Not applicable.

### Availability of data and materials

Data and materials are fully available and presented in the main paper.

## Abbreviations

CTV: clinical target volume
18F-FEC: F-18-Fluoroetyhlcholin
HIFU: high-intensity focused ultrasound
PCA: prostate cancer
PET/CT: positron emission tomography/computertomography
PSA: prostate-specific antigen
PSMA: prostate-specific membrane antigen
RTOG: Radiation Therapy Oncology Group
SPECT: single photon emission computed tomography
SUV max: maximum standardized uptake value
